# Rikkunshito, a Japanese Kampo Medicine, Ameliorates Decreased Feeding Behavior via Ghrelin and Serotonin 2B Receptor Signaling in a Novelty Stress Murine Model

**DOI:** 10.1155/2013/792940

**Published:** 2013-10-29

**Authors:** Chihiro Yamada, Yayoi Saegusa, Koji Nakagawa, Shunsuke Ohnishi, Shuichi Muto, Miwa Nahata, Chiharu Sadakane, Tomohisa Hattori, Naoya Sakamoto, Hiroshi Takeda

**Affiliations:** ^1^Tsumura Research Laboratories, Tsumura & Co., Ibaraki 300-1192, Japan; ^2^Pathophysiology and Therapeutics, Faculty of Pharmaceutical Sciences, Hokkaido University, Sapporo, Hokkaido 060-0812, Japan; ^3^Department of Gastroenterology and Hepatology, Hokkaido University Graduate School of Medicine, Sapporo, Hokkaido 060-8638, Japan; ^4^Department of Medical Gastroenterology, National Hospital Organization Hokkaido Medical Center, Sapporo, Hokkaido 063-0005, Japan

## Abstract

We investigated the effects of rikkunshito (RKT), a ghrelin signal enhancer, on the decrease in food intake after exposure to novelty stress in mice. RKT administration (500 mg/kg, *per os*) improved the decrease in 6 h cumulative food intake. In control mice, the plasma acylated ghrelin levels significantly increased by 24 h fasting. In contrast, the acylated ghrelin levels did not increase by fasting in mice exposed to the novelty stress. RKT administration to the novelty stress mice showed a significant increase in the acylated ghrelin levels compared with that in the distilled-water-treated control mice. Food intake after administering serotonin 2B (5-HT_2B_) receptor antagonists was evaluated to clarify the role of 5-HT_2B_ receptor activation in the decrease in feeding behavior after novelty stress. SB215505 and SB204741, 5-HT_2B_ receptor antagonists, significantly improved the decrease in food intake after exposure to novelty stress. A component of RKT, isoliquiritigenin, prevented the decrease in 6 h cumulative food intake. Isoliquiritigenin showed 5-HT_2B_ receptor antagonistic activity *in vitro.* In conclusion, the results suggested that RKT improves the decrease in food intake after novelty stress probably via 5-HT_2B_ receptor antagonism of isoliquiritigenin contained in RKT.

## 1. Introduction

Stress is becoming a significant social problem [1, 2] and is known to influence gastrointestinal function [3, 4]. One of the psychological stressors experienced in daily life is exposure to social environmental changes, but no detailed investigation regarding the effects of stress associated with this exposure on feeding behavior has been conducted. The novelty-induced hypophagia test measures the suppression of food intake by exposure to a novel environment and is one of the few animal tests of anxiety [5, 6].

Central 5-hydroxytryptamine (5-HT; serotonin) functions by evoking fear and anxiety manifestations and is involved in appetite regulation. Acute 5-HT depletion decreases anxiety behavior that is measured by inhibition of food intake during exposure to novel stimuli [7]. 5-HT_2C_ receptor (5-HT_2C_R) stimulation may decrease hunger [8, 9], and this type of receptor is expressed on corticotropin-releasing factor (CRF) neurons in the hypothalamic paraventricular nucleus and on proopiomelanocortin neurons in the arcuate nucleus [10]. In addition, stimulation of 5-HT_2B_Rs, which are distributed throughout the gastrointestinal system, negatively regulates eating behavior [11–13]. We previously confirmed that novelty stress decreases food intake by activating both CRF1R and 5-HT_2C_R [14]. However, we have been unable to clarify the role of 5-HT_2B_R activation in decreased food intake as a result of novelty stress.

Ghrelin is an orexigenic hormone produced in large quantities in the stomach [15]. Peripheral ghrelin binds to its specific growth hormone secretagogue receptor (ghrelin receptor) localized at the end of the vagus nerve around the stomach [16, 17]. Ghrelin secretion from the stomach is regulated by particular subtypes of some neurotransmitters. Activations of 5-HT_2B_R and 5-HT_2C_R lead to a reduction in the circulating ghrelin concentrations via a decrease in ghrelin secretion in the stomach [13]. In mice exposed to a novelty stress condition, plasma ghrelin levels decreased 3 h after stress application, and acylated ghrelin supplementation remedied this reaction [14].

Rikkunshito (RKT) is a Japanese Kampo medicine comprising ingredients that facilitate ghrelin signaling [13, 18, 19]. In gastrointestinal functional disorders, where 5-HT is excessively released, such as disorders following cancer chemotherapy [13] and SSRI administration [19], 5-HT_2B*⁄*2C_R stimulation causes decreased peripheral and central ghrelin concentrations, and RKT restores decreased peripheral acylated ghrelin secretion to normal levels via antagonizing these receptors. We have already demonstrated that RKT administration improves decreased food intake in novel environmental stress mice [14], but the underlying mechanism remains to be sufficiently elucidated.

We hypothesized that the improvement effects of RKT on decreased feeding behavior caused by novelty stress may be mediated through 5-HT_2B_R antagonism. To confirm this hypothesis, abnormal ghrelin dynamics in this stress model were clarified. In addition, to clarify the role of 5-HT_2B_R in the decreased food intake in novelty stress, the administration of 5-HT_2B_R antagonists was performed. Furthermore, we investigated the antagonistic effects of isoliquiritigenin, a component of RKT, on 5-HT_2B_Rs and its influence on food intake.

## 2. Materials and Methods

### 2.1. Chemicals

SB215505 (5-HT_2B_R antagonist) and SB204741 (5-HT_2B_R antagonist) were purchased from Sigma-Aldrich (St. Louis, MO, USA). All chemicals were dissolved in sterilized physiological saline before use. RKT was used as a powdered extract which was obtained by spray drying the hot water extract of a mixture of eight crude drug types: sojutsu (*Atractylodis lanceae rhizoma*), ninjin (*Ginseng radix*), hange (*Pinelliae tuber*), bukuryo (*Hoelen*), taiso (*Zizyphi fructus*), chinpi (*Aurantii nobilis pericarpium*), kanzo (*Glycyrrhizae radix*), and shokyo (*Zingiberis rhizoma*). RKT and RKT components were supplied by Tsumura & Co. (Tokyo, Japan).

### 2.2. Experimental Animals

Male C57BL/6J mice aged 6 weeks (Charles River Laboratories Japan, Inc., Tokyo, Japan) were used. Before the experiment, five mice per cage were maintained in a room with controlled temperature and humidity under a 07:00–19:00 light cycle with free access to food and water. For novelty stress, each mouse was transferred from group-housed cages to individual cages. Control mice were housed in individual cages for 7 days before the experiment was initiated. The mice in each group were similarly handled. All experiments were performed between 09:00 and 18:00 according to the guidelines established by the Experimental Animal Ethics Committee of Tsumura & Co. 

### 2.3. Food Intake

All protocols were performed under a 24 h fasting condition. Time-course evaluation of the effect of the novelty stress on food intake in 24 h fasted mice was undertaken 1, 2, 3, 6, and/or 24 h after exposure to the novelty stress, and the effect was calculated as the difference between the food weights before and after the feeding period at each time interval. 

**Figure 1:**
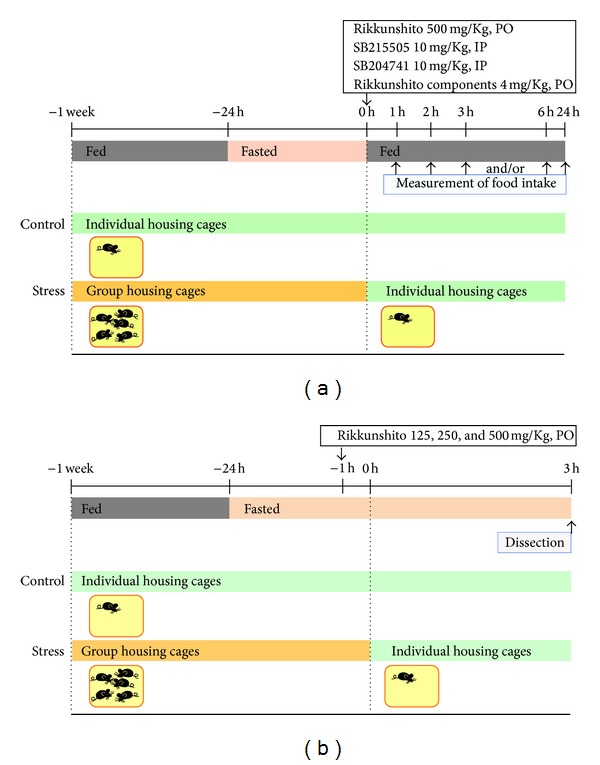
Experimental protocol. (a) Measurement of food intake. Cumulative food intake was measured at various time intervals after exposure to the novelty stress. (b) Measurement of plasma ghrelin and tissue mRNA levels. Blood, hypothalamus, and stomach were collected 3 h after exposure to the novelty stress.

To clarify the orexigenic action of RKT on food intake in stressed mice, we then investigated the effects of *per os* (PO) administration of RKT (500 mg/kg) [14] (Figure 1(a)) or RKT components (8-shogaol, nobiletin, tangeretin, glycyrrhizin, glycycoumarin, and isoliquiritigenin; 4 mg/kg, Figure 1(a)). The effect of intraperitoneal (IP) administration of SB215505 (10 mg/kg) or SB204741 (10 mg/kg) on the novelty stress-induced decrease in food intake was also investigated (Figure 1(a)). The experimental doses were chosen on the basis of a previous report [13]. RKT, RKT components, SB215505, or SB204741 was administered immediately after the onset of novelty stress.

### 2.4. Determining Plasma Levels of Ghrelin

To clarify the alteration of peripheral ghrelin dynamics after exposure to the novelty stress, blood was collected from mice given ether anesthesia 0.5 and 3 h after the novelty stress under the 24 h fasting and freely fed conditions. Blood collection to determine plasma ghrelin levels was performed from 10:00 to 12:00. We next investigated the effect of RKT (125, 250, or 500 mg/kg, PO) on plasma ghrelin concentration levels 3 h after exposure to the novelty stress (Figure 1(b)). RKT was orally administered 1 h before exposure to the novelty stress, and blood was collected 3 h after the exposure. The results of our evaluation of the postnovelty stress time course revealed that plasma ghrelin decreased significantly after 3 h [14]. We collected blood samples 3 h after stress to clarify the relationship between this change in plasma ghrelin levels and improved food intake.

The ghrelin levels were determined using commercial ELISA kit (Mitsubishi Chemical Medience Co., Tokyo, Japan).

### 2.5. Extraction of Total RNA for Reverse Transcription-Polymerase Chain Reaction (PCR)

The hypothalamus and stomach in mice treated with distilled water or RKT (500 mg/kg) 3 h after exposure to novelty stress were rapidly removed and immediately frozen by placing them in a tube on dry ice. Homogenization of the isolated tissue and total RNA extraction were performed according to the protocol from the RNeasy Universal Tissue Kit (Qiagen, Valencia, CA, USA), after which each sample was diluted to 100 ng/*μ*L. The diluted total RNA was incubated at 70°C for 5 min and then cooled on ice. A TaqMan Reverse Transcription Reagent kit (Applied Biosystems, Foster City, CA, USA) was used according to the manufacturer's protocol to reverse transcribe the total RNA (1000 ng). A TaqMan Universal PCR Master Mix (Applied Biosystems) was used to perform quantitative PCR assays with a Prism 7900HT Sequence Detection System (Applied Biosystems). To correct the differences in the amount of total RNA added to each reaction, ribosomal protein S29 (RPS29) as an endogenous control was used to normalize mRNA expression. These differences were expressed by the ΔCt (Ct: threshold cycle) value: ΔCt = 2^(−|*A*  −  *B*|)^, where *A* is the number of cycles that needed to reach the threshold for the housekeeping gene and *B* is the number of cycles needed for the target gene. All oligonucleotide primers and fluorogenic probe sets for TaqMan real-time PCR were manufactured by Applied Biosystems (RPS29: Mm02342448_gH, NPY: Mm00445771_m1, AgRP: Mm00475829_g1, preproghrelin: Mm00445450_m1, ghrelin receptor: Mm00616415_m1, orexin: Mm01964030_s1, leptin receptor: Mm00440174_m1, and CRF: Mm01293920_s1).

### 2.6. Binding Assay and Cell Function Assay for 5-HT_2B_R

CHO-K1 cells stably transfected with a plasmid encoding the human 5-HT_2B_R were used to prepare membranes in modified Tris-HCl buffer. A membrane protein was incubated with 1.2 nmol/mL [^3^H]LSD for 60 min at 37°C. Nonspecific binding was estimated in the presence of 10 *μ*mol/L serotonin. Membranes were filtered and washed, and then, the filters were assayed for radioactivity to determine the amount of specifically bound [^3^H]LSD [20]. 

The antagonistic activities of compounds on human 5-HT_2B_R expressed in transfected CHO-K1 cells were determined using the HTRF detection method to measure their effects on agonist-induced IP_1_ production [21]. Cells were suspended in 10 mM HEPES buffer pH 7.4, plated in 96-well microplates at a density of 4 × 10^4^ cells/well, and preincubated for 5 min at room temperature in the presence of the buffer (basal control) or the test compound. Thereafter, the reference agonist 5-HT was added at a final concentration of 30 nM. Separate assay wells did not contain 5-HT for basal control measurements. After a 30 min incubation at 37°C, the cells were lysed, and the fluorescence acceptor (D2-labeled IP_1_) and donor (anti-IP_1_ antibody labeled with europium cryptate) were added. After a 60 min incubation at room temperature, the fluorescence transfer was measured at *λ*_ex_ = 337 nm and *λ*_em_ = 620 and 665 nm. The IP_1_ concentration was determined by dividing the signal measured at 665 nm by that measured at 620 nm. Results were expressed as percent inhibition of the control response to 30 nM 5-HT. A concentration-response curve was generated to calculate the IC_50_ values.

### 2.7. Statistical Analysis

Statistical analyses of mean values of the two groups were performed using Student's *t*-test or Aspin-Welch's *t*-test after the *F*-test. Differences in multiple groups' mean values were assessed by Dunnett's analysis after Bartlett test. Cumulative food intake data were analyzed by repeated measures analysis of variance (ANOVA) followed by Dunnett's *post hoc* test. Data were expressed as the mean ± standard error of the mean (SEM) of each group, and *P* values < 0.05 were considered to indicate statistical significance.

## 3. Results

### 3.1. Changes in Food Intake in Mice Exposed to Novelty Stress

**Figure 2:**
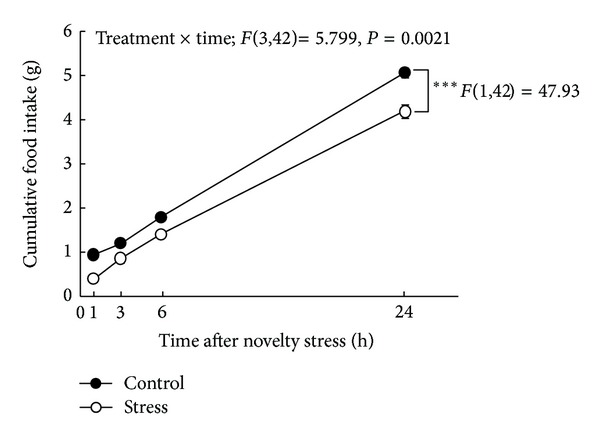
Changes in cumulative food intake after exposure to a novelty stress condition. Data are expressed as the mean ± SEM of 8 mice. ***P < 0.001 when analyzed by two-factor repeated measures ANOVA.

We investigated the effects of novelty stress on changes in food intake (Figure 2). Two-factor repeated measures ANOVA revealed that the effects of stress (*F*(1, 42) = 47.93, *P* < 0.001), time (*F*(3,42) = 1323, *P* < 0.001), and stress × time (*F*(3,42) = 5.799, *P* = 0.0021) were significant.

### 3.2. Effects of RKT on Food Intake in 24 h Fasted Mice

**Figure 3:**
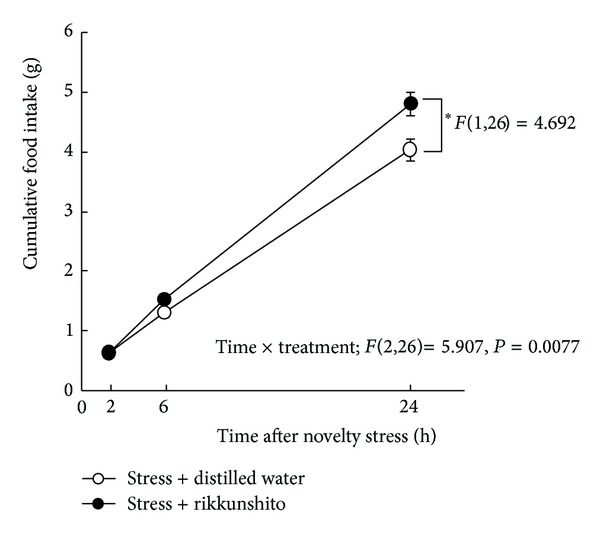
Effect of rikkunshito on food intake under a novelty stress condition. Data are expressed as the mean ± SEM of 7-8 mice. *P < 0.05 versus distilled-water-treated mice exposed to novelty stress conditions by two-factor repeated measures ANOVA.

To clarify the effects of RKT on food intake, RKT (500 mg/kg, PO) was administered to 24 h fasted mice. Food intake was measured 2, 6, and 24 h after exposure to novelty stress. RKT administration restored the decreased food intake significantly (*F*(1,26) = 4.692, *P* = 0.0495, Figure 3). Two-factor repeated measures ANOVA revealed that the effects of treatment × time (*F*(2,26) = 5.907, *P* = 0.0077) were significant.

### 3.3. Changes in Plasma Ghrelin Levels and Effects of RKT in Mice Exposed to Novelty Stress

**Figure 4:**
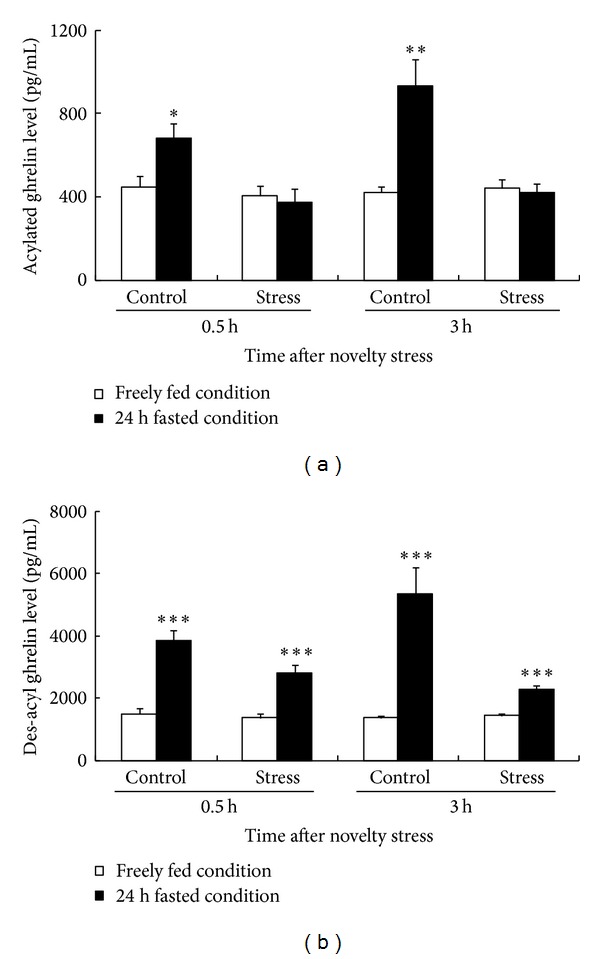
Changes in plasma ghrelin levels under the freely fed or 24 h fasted condition. (a) The plasma acylated ghrelin level. (b) The plasma des-acyl ghrelin level. Data are expressed as the mean ± SEM of 7-8 mice. *P < 0.05, **P < 0.01, and ***P < 0.001 versus mice fed freely by Student's *t*-test or Aspin-Welch's *t*-test.

We measured plasma ghrelin levels to clarify whether plasma acylated ghrelin played a role in decreasing food intake in the mice exposed to novelty stress. Plasma acylated ghrelin and des-acyl ghrelin levels at 0.5 and 3 h under ad libitum feeding were not significantly different between the control and novelty stress groups. Under the 24 h fasting condition, plasma acylated ghrelin levels in the stress group were not significantly increased compared with those under ad libitum feeding; however, the des-acyl ghrelin level significantly increased, while still being lower than that in the control group (0.5 h; *P* < 0.001, 3 h; *P* < 0.001, Figure 4). 

**Figure 5:**
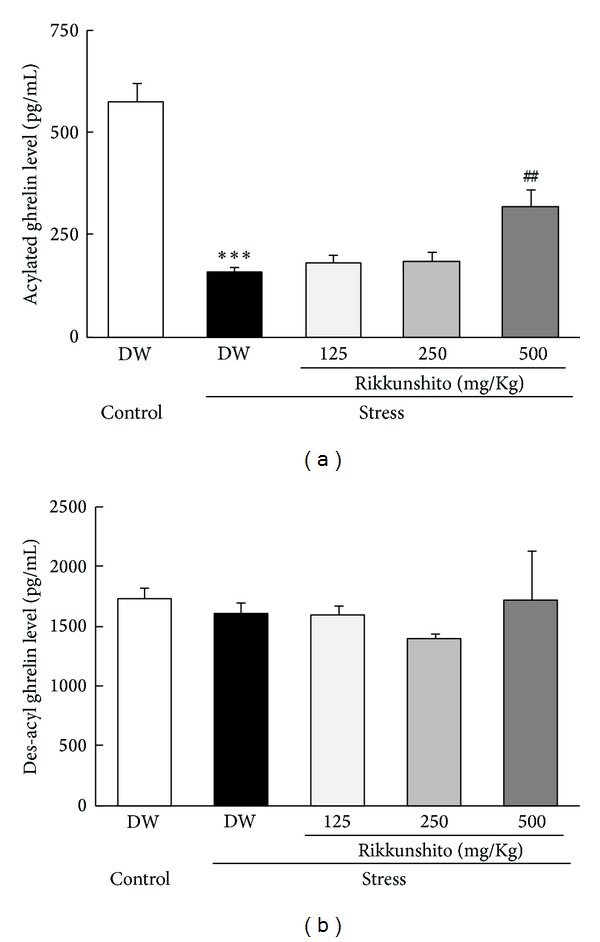
Effect of rikkunshito on plasma ghrelin levels in mice exposed to novelty stress. Plasma ghrelin levels were determined 3 h after onset of novelty stress. (a) Plasma acylated ghrelin level. (b) Plasma des-acyl ghrelin level. Data are expressed as the mean ± SEM of 8 mice. ***P < 0.001 versus control group by Aspin-Welch's *t*-test and ^##^ *P* < 0.01 versus the distilled-water-treated mice exposed to stress by Dunnett's analysis. DW: distilled water. The partial data of acylated ghrelin level indicated in this figure are derived from [14].

Next, we detected the effects of RKT administration on plasma ghrelin levels under the 24 h fasted condition. RKT administration prevented a decrease in plasma acylated ghrelin levels compared with distilled water 3 h after exposure to novelty stress (*P* = 0.0074, Figure 5). The RKT-treated mice showed a trend toward increased plasma des-acyl ghrelin levels compared with the distilled water-treated stress mice, but the difference was not significant.

### 3.4. Effects of RKT on mRNA Expression of Orexigenic Factors in Mice Exposed to Novelty Stress

**Figure 6:**
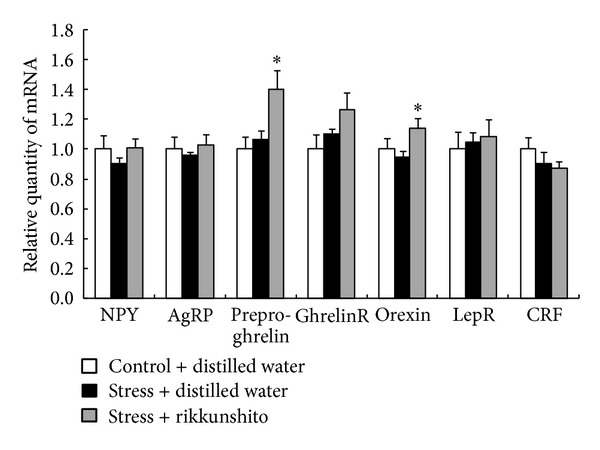
Effects of rikkunshito on hypothalamic appetite-related factor gene expression in mice exposed to a novelty stress condition. The hypothalami were collected after a 3 h exposure to novelty stress condition (4 h after rikkunshito 500 mg/kg, PO). The data are expressed as the mean ± SEM of 8 mice. *P < 0.05 versus distilled-water-treated mice exposed to a novelty stress condition by Student's *t*-test or Aspin-Welch's *t*-test.

To clarify the effects of RKT on gene expression of orexigenic factors after exposure to novelty stress, we evaluated this effect on hypothalamic and gastric mRNA expression 3 h after exposure. In the stress group, hypothalamic NPY and AgRP mRNA showed a trend toward decreased mRNA expression. RKT administration (500 mg/kg, PO) showed a tendency to increase NPY and AgRP mRNA expression compared with stress, but the difference was not statistically significant (Figure 6). Preproghrelin gene expression was significantly increased by RKT treatment (*P* = 0.036), although this remained unchanged 3 h after novelty stress. Levels of ghrelin receptor mRNA in the RKT-treated mice showed an increasing trend, but the difference was not statistically significant. Orexin mRNA expression in the RKT-treated mice was significantly different from that in the distilled-water-treated stress mice (*P* = 0.023). There were no significant changes in leptin receptor and CRF mRNA expression among all groups. In addition, there were no significant changes in gastric preproghrelin mRNA expression among all groups (control group, 1.0 ± 0.03; novelty stress group, 1.1 ± 0.02; stress + RKT group, 1.1 ± 0.03 relative quantity of mRNA; data are not shown in figures and tables). 

### 3.5. Effects of 5-HT_2B_R Antagonists on Food Intake

**Figure 7:**
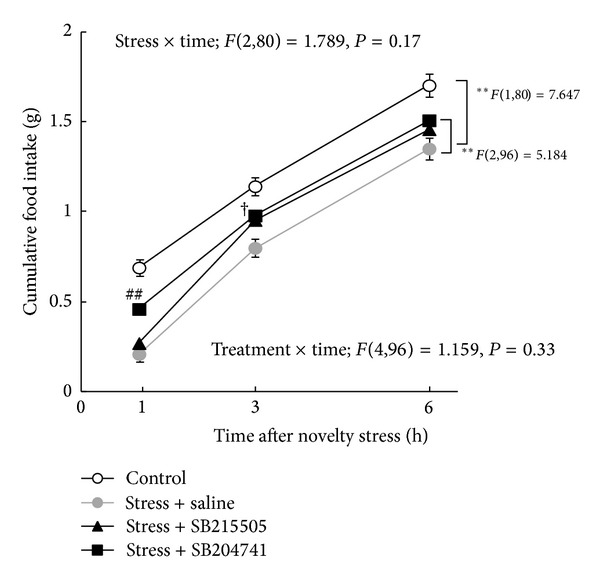
Effects of SB215505 or SB204741, 5-HT_2B_ receptor antagonists, on cumulative food intake in mice exposed to a novelty stress condition. Data are expressed as the mean ± SEM of 10–21 mice. **P < 0.01 when analyzed by two-factor repeated measures ANOVA. ^##^ *P* < 0.01 SB204741 treatment versus saline-treated mice exposed to novelty stress by Dunnett's *post hoc* analysis. ^†^ *P* < 0.05 SB215505 treatment versus saline-treated mice exposed to novelty stress by Dunnett's *post hoc* analysis.

Cumulative food intake was decreased in the group exposed to novelty stress compared to control mice in the first 6 h after exposure to the stress (*F*(1,80) = 7.647, *P* = 0.0086, Figure 7). Two-factor repeated measures ANOVA revealed that the effects of stress × time were not significant (*F*(2,80) = 1.789, *P* = 0.17). Administration of a 5-HT_2B_R antagonist (SB215505; 10 mg/kg, IP or SB204741; 10 mg/kg, IP), significantly ameliorated the decrease in food intake (*F*(2,96) = 5.184, *P* = 0.0092). Administration of SB215505 significantly prevented the decrease in food intake 3 h after exposure to novelty stress (*P* = 0.044), whereas SB204741 prevented the decrease in food intake for 1 h after exposure to stress (*P* = 0.0015).

### 3.6. Effects of Herbal RKT Components on Food Intake

**Figure 8:**
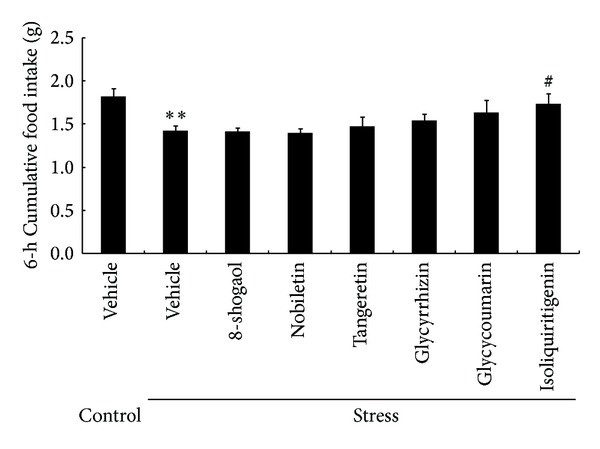
Effects of rikkunshito components on 6 h food intake in mice exposed to a novelty stress condition. Data are expressed as the mean ± SEM of 8–16 mice. **P < 0.01 versus control mice by Student's *t*-test and ^#^ *P* < 0.05 versus vehicle- (0.5% carboxymethylcellulose-) treated mice exposed to a novelty stress condition by Dunnett's analysis.

We investigated the effects of six components of RKT on decreased food intake after exposure to novelty stress. The 6 h cumulative food intake was significantly decreased in the novelty stress-exposed mice compared with that in the control mice (*P* = 0.0012, Figure 8). Isoliquiritigenin administration (4 mg/kg, PO) prevented a decrease in cumulative food intake (*P* = 0.045). Glycycoumarin (4 mg/kg, PO) administration showed a tendency to alleviate decreased food intake in stressed mice, although the effect was not statistically significant. The other RKT components investigated exerted no effects on decreased food intake.

### 3.7. IC_50_ Values for 5-HT_2B_R

Table 1 shows the 5-HT_2B_R-binding inhibitory and cell function activities of isoliquiritigenin contained in RKT. Isoliquiritigenin showed an IC_50_ for 5-HT_2B_R binding of 6.3 ± 0.0 *μ*mol/L and an inhibitory cell function activity of 2.1 ± 0.2 *μ*mol/L.

## 4. Discussion

In this study, we demonstrated that the novelty stress decreased food intake and suppressed a physiological increase in plasma acylated ghrelin levels after fasting in mice. Oral RKT administration significantly suppressed this novelty-induced hypophagia and decrease in acylated ghrelin levels during fasting. Furthermore, decreased food intake caused by the novelty stress was significantly suppressed by 5-HT_2B_R antagonists and isoliquiritigenin, an ingredient of RKT which has 5-HT_2B_R antagonistic activity *in vitro* that exhibits the same effect.

The effects of drugs on anxiety responses in animal models are generally evaluated by the open field test in novelty environments [22]. In addition, novelty stress models are one of the established methods for evaluating feeding behavior, and novel environmental research using decreased food intake as an index has previously been conducted [7, 23]. Using this methodology, we reported that the acute novel environmental change caused by conversion from group housing to individual housing significantly suppressed feeding behavior in both young [14] and aged mice [24].

During fasting, secretion of acylated ghrelin by the stomach was enhanced, increasing the circulating levels [25]. However, in this study, no increase was observed in the fasting plasma levels of acylated ghrelin in mice exposed to novelty stress. In a previous study, it was clearly demonstrated that exogenous acylated ghrelin supplementation negated decreased food intake in the same model as that used in this study [14]. These results and findings suggest that the transmission of ghrelin signals to the hypothalamic feeding center under the fasting condition is decreased during stress responses caused by novel environmental changes. Although plasma des-acyl ghrelin (a metabolite of acylated ghrelin) levels after 24 h of fasting were significantly enhanced in mice exposed to novelty stress, the intensity of the increase was much lower than that in the nonstress-exposed mice. In addition, novel environmental changes did not cause any significant changes in the expression of gastric preproghrelin or ghrelin-*O*-acyltransferase gene (data are not shown). Therefore, novel environmental stress suppresses the secretion of acylated as well as des-acyl ghrelin, whereas it does not affect the biosynthesis of acylated ghrelin in the stomach during fasting. 

NPY and AgRP gene expression in the hypothalamus tended to decrease in the novel environmental change group relative to that in the control group during the 3 h after stress exposure, although no statistically significant difference was observed. Ghrelin is secreted from X/A-like cells in the gastric mucosa and acts on ghrelin receptors in vagus nerve endings, then activating NPY/AgRP neurons in the hypothalamic arcuate nucleus via the vagus nerve [16]. The results of this study did not convincingly verify the attenuation of ghrelin signaling caused by stress when upregulation of hypothalamic NPY/AgRP gene expression was used as an index for ghrelin signaling. The reason why NPY and AgRP mRNA expression were not significantly decreased in mice exposed to novelty stress is unknown. With regard to NPY, there may be interference from mRNA expression in hypothalamic tissue other than the arcuate nucleus. To this end, the *in situ *hybridization technique is required for accurate evaluation of NPY and AgRP mRNA in the arcuate nucleus.

RKT, a Japanese Kampo medicine, is known to increase levels of peripheral acylated ghrelin in humans [26], rodents [13, 27], and dogs [28] as well as increase hypothalamic acylated ghrelin in rodents [29]. RKT also enhances the binding of ghrelin to ghrelin receptors [18, 30], resulting in enhanced and prolonged ghrelin signaling. We have previously reported that RKT administration to mice exposed to novelty stress suppresses a reduction in food intake 1 and 3 h after isolation, and the effects by RKT are abolished by coadministration of RKT with a ghrelin receptor antagonist [14]. The present data regarding the effect of RKT on food intake is almost in agreement with previous findings. In our experiment, RKT significantly reversed the decrease in peripheral acylated ghrelin levels caused by exposure to novelty stress. In contrast, no obvious effect of RKT was observed on des-acyl ghrelin levels after stress. On the basis of these results, we conclude that the increase in peripheral acylated ghrelin level associated with RKT may be mediated through enhanced acylated ghrelin secretion [13] in addition to the inhibition of acylated ghrelin metabolism [27].

In this study, enhanced hypothalamic preproghrelin and orexin mRNA expression and a tendency toward increased ghrelin receptor mRNA expression were observed following RKT administration. Activation of orexin neurons occurs downstream in the ghrelin signaling pathways, and the signal to increase appetite is transmitted to higher-order neurons via orexin gene expression. Enhancement of orexin mRNA by RKT may suggest ghrelin signal-enhancing effects. In addition, RKT promoted the secretion of ghrelin in the hypothalamus in cisplatin-induced hypophagia models [29] and ghrelin receptor gene expression [30]. These results may be supported by other studies indicating enhanced expression of preproghrelin mRNA [26] and ghrelin receptor mRNA [30], despite differences in models. In addition, no significant changes in the expression of these genes can be confirmed in mice exposed to novelty stress. Our results were obtained 3 h after exposure to stress, but because hypophagia was actually observed 1 h after exposure, it may be necessary to reexamine sampling times.

It is well known that a stress model exhibits higher levels of central 5-HT and expression of 5-HTR, leading to activation of the serotonergic signal [6, 24]. We previously established the involvement of central 5-HT_2C_R activation in decreased food intake during exposure to novel environmental stress and demonstrated that abnormalities in ghrelin dynamics may partially contribute to this reaction [14]. Contrary to 5-HT_2C_Rs, 5-HT_2B_Rs are sparsely expressed in discrete subregions of the central nervous system (CNS) [31], whereas they are heavily expressed in the periphery [32]. In a stomach, 5-HT_2B_Rs are distributed throughout the gastric submucosa and smooth muscle, and their activation is known to result in contraction of the gastric fundus strip [33]. Although there have been several reports on the association between stress and 5-HT in gastrointestinal organs [34, 35], direct relationship between peripheral 5-HTR activation and novelty stress has not been proven. In the current study, we found that 5-HT_2B_R antagonism inhibited the decrease in food intake after novelty stress. Because there is no information available on the 5-HT_2B_R antagonists used in this study in terms of blood-brain barrier permeability, we could not determine whether 5-HT_2B_R antagonism was effective in the CNS or the peripheral in the present study. Further investigation is required to determine the 5-HT_2B_R activating site under stress.

5-HT_2B_R activation by peripheral administration of a 5-HT_2B_R agonist has been shown to cause a decrease in food intake [12] and inhibition of ghrelin secretion [13]. We also found that peripheral administration of BW723C86, a 5-HT_2B_R agonist, decreased plasma acylated and des-acyl ghrelin levels in normal rats (see Supplemental Material available online at http://dx.doi.org/10.1155/2013/792940). These findings suggest that 5-HT_2B_R activation is associated with abnormal ghrelin dynamics.

In the present study, the effects of certain RKT components on food intake in stress models were examined. Administration of isoliquiritigenin (4 mg/kg) significantly improves novelty stress-induced hypophagia. We also found that isoliquiritigenin inhibited binding between 5-HT and 5-HT_2B_R and confirmed that it has an obvious antagonistic effect on 5-HT_2B_R using a cell function assay. We previously reported that glycycoumarin, which has an antagonistic effect on 5-HT_2B_R [13], suppressed decreased food intake 3 h after the application of stress [14]. It seems likely that glycycoumarin may inhibit the decrease in food intake after exposure to novelty stress via 5-HT_2B_R antagonism. Therefore, multiple RKT ingredients having an antagonistic effect on 5-HT_2B_R act and are considered to be responsible for its effects.

## 5. Conclusion

In conclusion, RKT has an ameliorating effect on decreased food intake caused by novel environmental changes. This effect appears to be mediated through improvement of abnormal ghrelin dynamics by 5-HT_2B_R antagonism.

## Supplementary Material

Effect of BW723C86, a 5-HT_2B_ receptor agonist on plasma acylated and des-acyl ghrelin level. A) The plasma acylated ghrelin level. B) The plasma des-acyl ghrelin level. BW723C86 (16 mg/kg, IP) was administered to rats and blood samples were collected 60 min after the treatment by decapitation. The control rats was administered saline IP. Data are expressed as the mean ±SEM of 8 rats. ^*∗*^P < 0.05; ^*∗∗*^P < 0.01 vs. control by Student's *t*-test or Aspin-Welch's *t*-test.

## Figures and Tables

**Table 1:** The inhibitory activity for 5-HT_2B_ receptor.

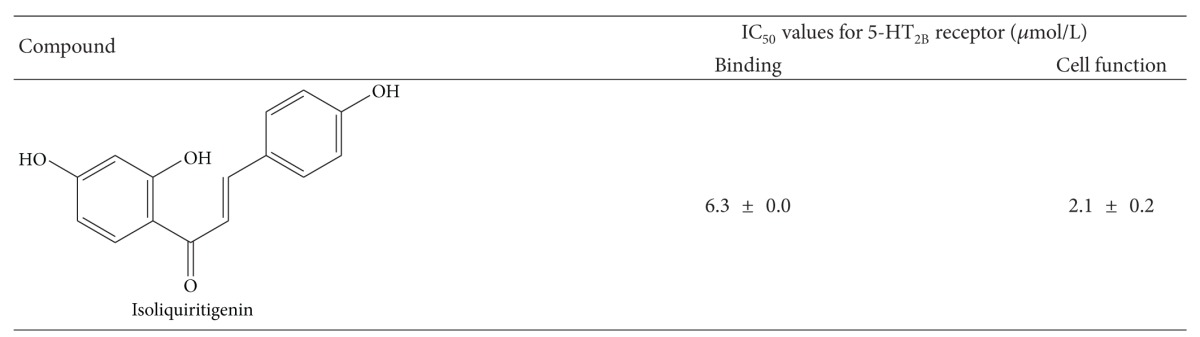

Each value indicated the mean ± SEM of 3 samples.
