# Supplementary material for: Rikkunshito, a Japanese Kampo Medicine, Ameliorates Decreased Feeding Behavior via Ghrelin and Serotonin 2B Receptor Signaling in a Novelty Stress Murine Model
Source: Biomed Res Int. 2013 Oct 29;2013:792940. doi: 10.1155/2013/792940 (PMC3830778; doi:10.1155/2013/792940)
Supplement: Supplementary file 1 — Effect of BW723C86, a 5-HT2B receptor agonist on plasma acylated and des-acyl ghrelin level. A) The plasma acylated ghrelin level. B) The plasma des-acyl ghrelin level. BW723C86 (16 mg/kg, IP) was administered to rats and blood samples were collected 60 min after the treatment by decapitation. The control rats was administered saline IP. Data are expressed as the mean ±SEM of 8 rats. ∗P < 0.05; ∗∗P < 0.01 vs. control by Student's t-test or Aspin-Welch's t-test. [file 792940.f1.pdf]

SUPPLEMENTAL MATERIAL:

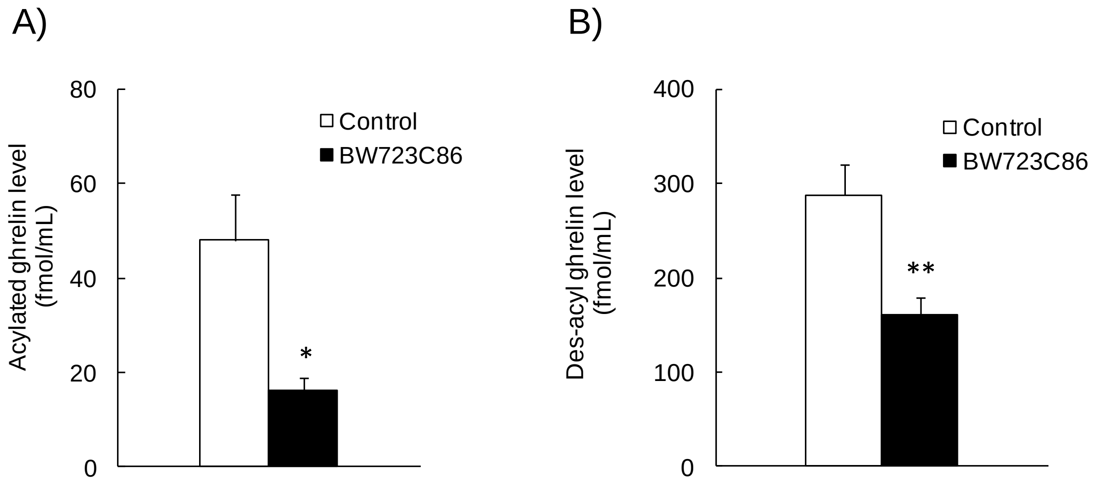

Supplemental material

Effect of BW723C86, a 5-HT$_{2B}$ receptor agonist on plasma acylated and des-acyl ghrelin level.
A) The plasma acylated ghrelin level. B) The plasma des-acyl ghrelin level. BW723C86 (16 mg/kg, IP) was administered to rats and blood samples were collected 60 min after the treatment by decapitation. The control rats was administered saline IP. Data are expressed as the mean ± SEM of 8 rats. *, $P < 0.05$; **, $P < 0.01$ vs. control by Student's *t*-test or Aspin-Welch's *t*-test.
